# Community pharmacist counseling improves adherence and asthma control: a nationwide study

**DOI:** 10.1186/s12913-022-07518-0

**Published:** 2022-01-26

**Authors:** Barbara Putman, Louise Coucke, Anna Vanoverschelde, Els Mehuys, Lies Lahousse

**Affiliations:** Pharmaceutical Care Unit, Faculty of Pharmaceutical Sciences, Ghent University, Ottergemsesteenweg 460, B-9000 Ghent, Belgium

**Keywords:** Asthma clinical care, Adherence to therapy, Asthma control, Asthma pharmacotherapy, Patient education and asthma

## Abstract

**Background:**

Pharmaceutical counseling (PC) interventions have been shown to improve adherence to controller medication and asthma control. However, the real-life impact of these PC interventions in difficult-to-control asthma patients remains unclear. We aimed to assess the effectiveness of PC interventions in real life using nationwide claims data.

**Methods:**

Demographics and drugs use of patients who received ICS in 2017 with or without pharmaceutical counseling were retrieved from a Belgian claims database. Asthma-related drug use from 1 year before first ICS dispensing in 2017 (reference period) was compared with 1 year after. Outcomes were usage of inhaled corticosteroids (ICS) in defined daily doses (DDD), proportion of users of short-acting beta-agonist (SABA), antibiotics, oral corticosteroids (OCS), asthma biologicals and controller-to-total (CTT) ratio.

**Results:**

The study population consisted of difficult-to-control asthma patients aged 5–40 years with at least the first interview within 90 days after first ICS dispensing (*n* = 1350). ICS usage increased significantly in the year after PC intervention compared with the reference period (+ 43.3 DDD/patient, *p* < 0.05). A nominal decrease was observed in the proportion of SABA (48.0 to 46.2%) and antibiotics (54.5 to 52.7%) after PC intervention compared with the reference period. CTT ratio significantly increased from 0.671 to 0.749 (*p* < 0.05). The proportion of biological users was nominally lower in the intervention group compared with a control group (*n* = 50,477) in the post-intervention time period (0.22% versus 0.30%).

**Conclusions:**

This first nationwide study among difficult-to-control asthma patients suggests that community pharmacist counseling is effective in real life to improve controller adherence and asthma control.

**Supplementary Information:**

The online version contains supplementary material available at 10.1186/s12913-022-07518-0.

## Background

Asthma is a prevalent chronic airway disease, often starting during childhood and affecting individuals of all ages [1]. Its worldwide prevalence was around 300 million individuals in 2016 according to the World Health Organization, with an estimated rise to 400 million expected by 2025 [2]. In Belgium, the prevalence is around 7% [3]. Despite the wide range of adequate asthma medication available, only 50% of patients benefit sufficiently from it. Inhaled corticosteroids (ICS) are the cornerstone of asthma therapy [4, 5]. However, adherence to controller medications and inhaler technique, remains suboptimal [6]. Uncontrolled asthma can lead to exacerbations, disability, and even death, resulting in a large socio-economic burden for the community. Therefore, asthma requires global attention and appropriate patient education, to improve patient outcomes [7].

Pharmacists may improve patient outcomes through pharmaceutical counseling (PC), which involves an interaction between pharmacist and patient with the objective to improve medication use by teaching them about disease and drug application [8]. A pre-defined PC intervention has shown to improve the adherence to controller medication, leading to improved asthma control of insufficiently controlled patients in a 6-month randomized, controlled trial in Belgian asthma patients [9]. In addition, a recent meta-analysis based on nine RCTs confirmed that PC interventions can effectively contribute to improved medication adherence in adult asthma patients [10], while another meta-analysis based on only two RCTs of asthma patients could not confirm improved adherence in this subgroup [11]. Most studies had a follow-up time of 6 months and included patients with mixed levels of asthma control [10]. However, data on the real-life impact of these PC interventions are lacking, particularly in difficult-to-control and severe asthma patients, a group who are associated with substantial health and economic burden [12, 13].

The overall aim of this study was to evaluate whether PC interventions improve adherence to chronic inhaler therapy among difficult-to-control asthma patients using real-world, nationwide data. We hypothesized that PC interventions improve ICS adherence and improved adherence results in a better asthma control.

## Methods

The source was the BelPhar database, which collects monthly reimbursement claims and patients’ demographics from all community pharmacies affiliated with the Association of Pharmacists Belgium (APB; the Belgian federation of independent community pharmacies). At national level, the registered data represents around 85% of all Belgian community pharmacies and corresponds to approximately 78% of all national reimbursed community pharmaceutical dispenses. The database from these community pharmacies open to the general public contains information on dispensed and reimbursed medicines, magisterial preparations, and various fees for community pharmacist services (in the context of guard duties, oxygen delivery, or care programs for chronic complex diseases including asthma, diabetes or chronic renal insufficiency). In Belgium, respiratory medicines listed in Additional Table 1 are on prescription and reimbursed.

**Fig. 1:**
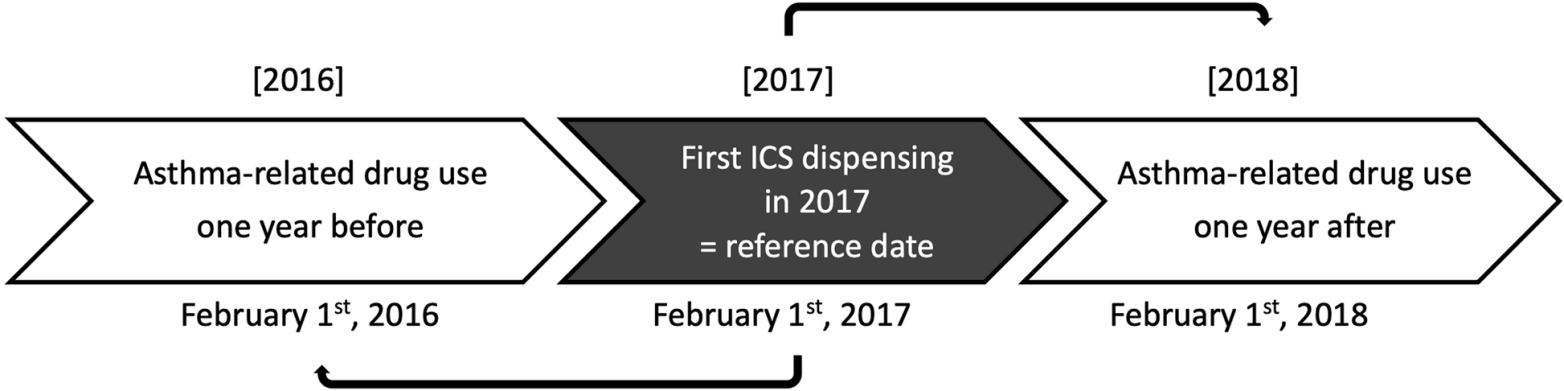
Example of data extraction. An example of how the BelPharData was extracted for a fictional patient receiving the first ICS dispensing in 2017 on February 1st, 2017 (= reference date). The asthma-related drug history 1 year before ICS dispensing (February 1st, 2016) was observed in comparison to the drug use exactly 12 months after the reference date

The source population contained all patients aged 5+ in the BelPharData with at least one ICS dispensing in the period 1/1/2017–31/12/2017, with the date of first dispensing in 2017 defining the reference date. The asthma-related drug use (detailed in Additional Table 1) of this population was extracted from exactly 1 year before to exactly 1 year after the reference date. The method used for the data extraction is illustrated in Fig. 1. Chronic ICS users were defined as patients in the source population who had at least one ICS dispensing in the 12 months before and in the 12 months after the reference date. We assumed that for non-chronic indications that required ICS treatment, the duration of the ICS treatment was shorter than 12 months. The inclusion criteria for the study population were chronic ICS users aged 5–40 years, considered as most representative of an asthmatic population. Patients aged over 40 years were not our target population since in this age group a higher prevalence of ICS usage includes many patients with COPD or those with both asthma and COPD [4]. Index patients received at least one PC intervention within 90 days after ICS dispensing, as recorded by the database.

**Fig. 2:**
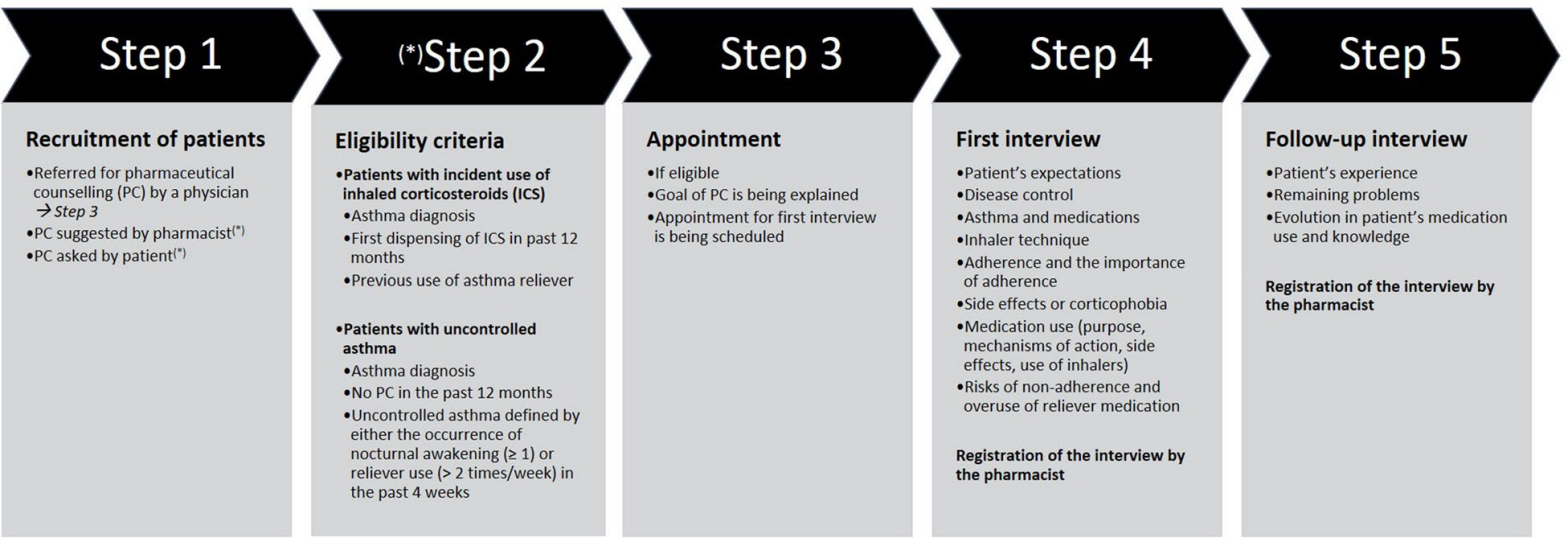
Pharmaceutical counseling (PC) intervention protocol. *Step 2 in case PC is suggested by pharmacist or asked by patient

Recruitment of the patients was possible if a PC intervention was 1) prescribed by a physician, 2) suggested by the pharmacist or, 3) at request of the patient (Fig. 2). The protocol-based PC intervention consisted of a first interview (Fig. 2 -Step 4) and a follow-up interview (Fig. 2 Step 5) offered by the community pharmacist during Belgian routine practice [14] and targeted both incident asthma patients starting ICS treatment and prevalent asthma patients with difficult-to-control asthma, which was defined as uncontrolled disease despite the prescription of asthma treatment [15]. Our study population was derived from this latter group with difficult-to-control asthma prescribed long-term ICS therapy, who received a PC intervention. Asthma control was assessed (Fig. 2 -Step 2) by the occurrence of nocturnal awakening (‘how often did you wake up at night or early in the morning earlier than usual because of asthma symptoms?’) and reliever use (‘how often have you used your inhaler with fast-acting medication?’) in the past 4 weeks, according to GINA guidelines [4]. In case of any nocturnal awakening in the past 4 weeks and/or reliever use more than twice a week, the asthma was considered difficult-to-control and an interview was scheduled at the community pharmacy (Fig. 2 -Step 3). In a first interview the pharmacist assessed patients’ expectations, disease control using Asthma Control Test [16], knowledge about asthma and medications, inhaler technique, adherence and the importance of adherence, side effects or corticophobia (Fig. 2 -Step 4). Furthermore, the patient was educated about medication use (purpose, mechanisms of action, side effects, use of inhalers) and risks of non-adherence and overuse of reliever medication, and possible questions were answered. A follow-up interview was offered three to 6 weeks later, to assess patient’s experience, detect remaining problems, and check the evolution in patient’s medication use and knowledge (Fig. 2 -Step 5). Belgian pharmacists receive a fee of about 20 euros per interview once every year for the PC service from the National Institute for Health and Disability Insurance, provided that the patient meets the reimbursement conditions, by registering the patient’s interview and date [14]. Through the flagging of the fee, we were able to capture patients with a PC intervention in the BelPhar database. A sensitivity analysis stratified on the number of PC interventions compared ICS usage between patients who received only the first interview (*n* = 1119, 83%)) with patients who received also a follow-up interview (*n* = 231, 17%). In both groups, we compared the drug use of the year after first ICS dispensing in 2017 with the drug use the year before. Also, in both groups the first interview happened within 90 days after ICS dispensing.

The change in drug use from 1 year before to 1 year after the PC intervention was analyzed. Asthma-relevant drug use was the main outcome of interest, reported as drug usage for ICS and proportion of drug users for the other asthma medications. Drug usage for ICS was defined as the proportion of total Defined Daily Dose (DDD) of ICS and the number of patients receiving ICS. Proportion of drug users was defined as the number of patients receiving an asthma-related drug of interest divided by the total number of ICS patients. For SABA, antibiotics and OCS, the proportion drug users from 1 year before first ICS dispensing in 2017 (reference period) was compared with 1 year after. The ratio of controller-to-total (CTT) asthma medications, an overall proxy for adherence and asthma control, was derived from the reported drug use [17–19]. The CTT ratio was calculated by dividing the sum of prescription DDDs for controller medication (ICS, long-acting beta-agonists [LABA], long-acting muscarinic antagonists and biologicals) by total asthma medication (controller medication plus SABA). Since omalizumab was the only reimbursed asthma biological available on the Belgian market until the end of 2016 and more became available during 2017, we only analyzed the proportion of biological users post PC intervention. Controls for this analysis were defined as chronic ICS users aged 5–40 years who did not receive a PC intervention (*n* = 50,477).

Confidence intervals for the group means were computed by the assumed mean method. Confidence intervals for the difference between two proportions were calculated using the Newcombe method [20]. *P* value < 0.05 was considered significant. Data analyses were performed using SAS 9.4 (SAS Institute Inc. Cary, NC). Figures were made using Microsoft Excel 16.45.

## Results

The BelPharData-based source population included 922,943 patients having an ICS dispensing recorded in 2017. Of this source population, 288,069 (31%) were chronic ICS users, of whom 56,582 (20%) were aged 5 to 40 years. Among these 5-to-40-year-old asthma patients on chronic ICS therapy, about half (54%) were women and 6% received at least one pharmaceutical counselling interview. Furthermore, 49% were SABA users, 51% were antibiotic users and 8% OCS users. The average ICS adherence was 36%, based on their DDD coverage in 2017. The study population consisted of difficult-to-control asthma patients identified by the pharmacist in this group of chronic ICS users aged 5–40 years, having their first interview within 90 days after the reference date (*n* = 1350). Among those, 83% (*n* = 1119) received only the first interview and 17% (*n* = 231) received also a follow-up interview.

### Impact on asthma controller medication

**Fig. 3:**
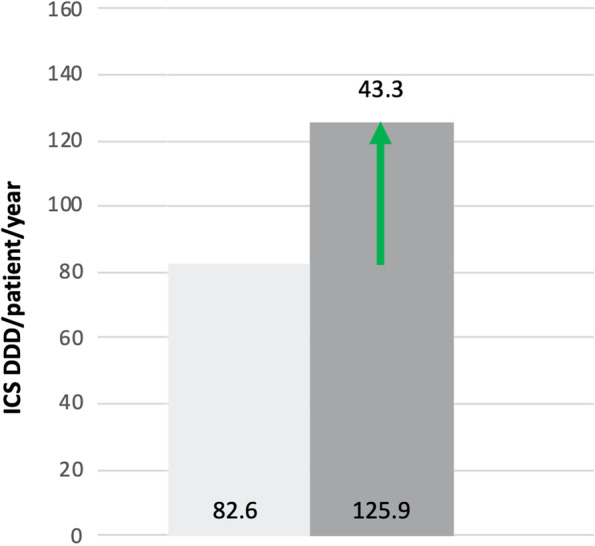
Inhaled corticosteroids (ICS) usage pre- and post-intervention. Bar chart showing results of the primary analysis of ICS usage 1 year pre-intervention (light gray filled) and 1 year post-intervention (dark gray filled) in 5–40 year-old difficult-to-control asthma patients with at least one PC intervention in 2017

The primary analysis showed a significant increase of 43.3 DDD/patient in ICS usage with 125.9 DDD/patient (95% Confidence Interval [CI] 124.9–126.9) in the year following the PC intervention compared with a usage of of 82.6 DDD/patient (95% CI 82.0–83.2) the year before (Fig. 3). The CTT ratio went from 0.671 before to 0.749 after PC intervention, representing an increase of 0.078 (95% CI 0.075–0.081, *p* < 0.05).

A sensitivity analysis on the number of interventions showed a similar, significant increase in ICS usage in patients who received only the first interview (*n* = 1119; 83%) of 39.3 DDD/patient (Additional Fig. 1). Patients who also received a follow-up interview (*n* = 231; 17%) showed a significant gain of even greater magnitude in ICS usage of 62.1 DDD/patient.

### Impact on short-acting beta-agonist (SABA), antibiotic and oral corticosteroid (OCS) use

**Fig. 4:**
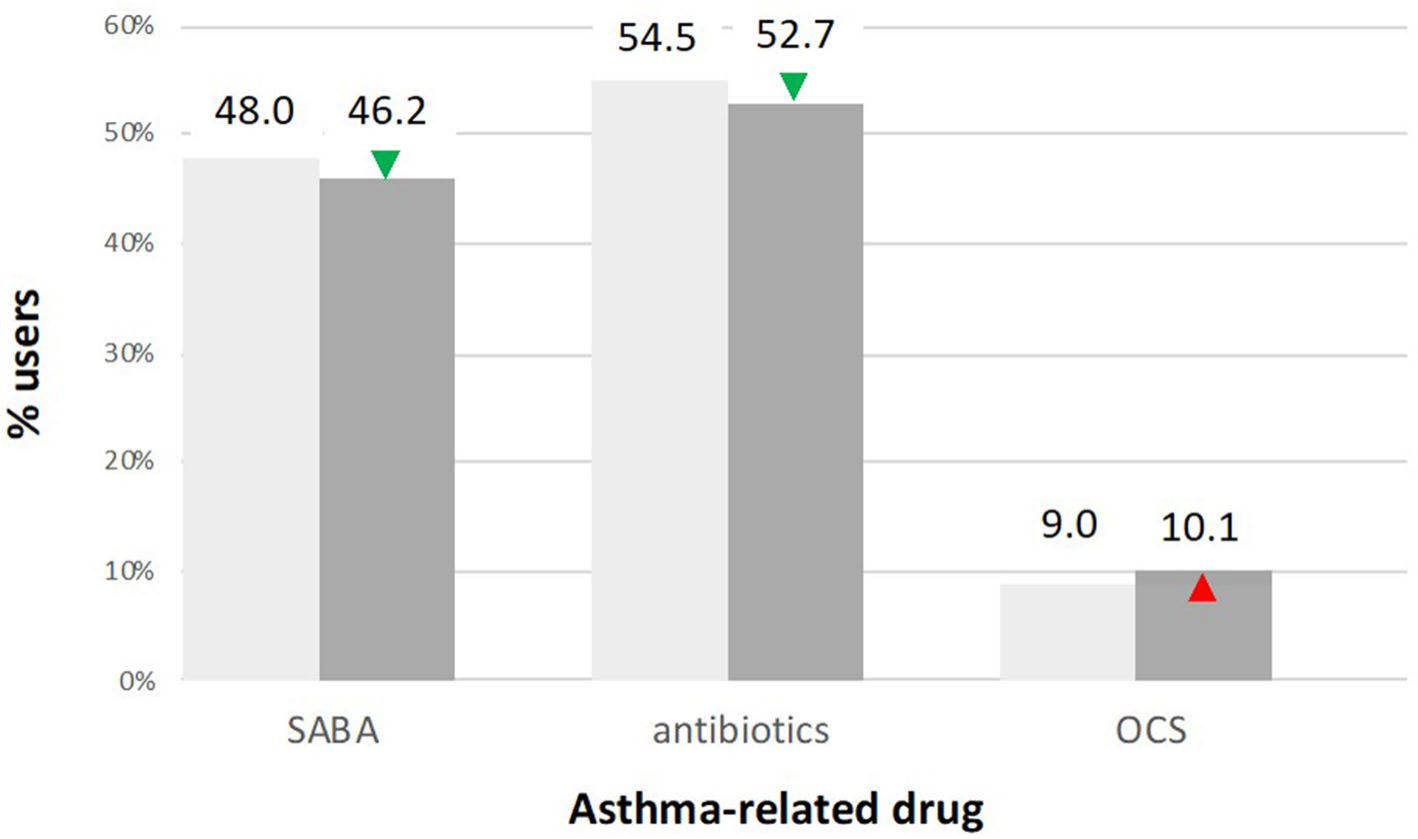
Use of asthma-related drugs pre- and post-intervention. Bar charts showing the proportion of users of asthma-related drugs among difficult-to-control 5–40-year-old asthma patients who received at least one PC intervention in 2017 (*n* = 1350). The left bars represent the percentage (top) with number (bottom, italics) of short-acting beta-agonist (SABA) users pre-(light gray filled) to post-(dark gray filled) PC intervention, the middle bars the percentage antibiotic users and the right bars the percentage oral corticosteroid (OCS) users

Difficult-to-control 5–40-year-old asthma patients who received a PC intervention in 2017 experienced a reduction in reliever use. The proportion of SABA users decreased from 48.0% in the year before the intervention to 46.2% in the year after the intervention, leading to a 1.8% (95% CI -2.0, 5.5%; *p* > 0.05) lower proportion of SABA users (Fig. 4). The proportion of antibiotic users was pronounced with 54.5% in the reference period. After the intervention a proportion of 52.7% was observed, leading to a 1.8% (95% CI -2.0,5.5%; *p* > 0.05) lower proportion of antibiotic users compared with the reference period. Regarding the OCS use, there was a 1.1% (95% CI -1.1,3.3%; *p* > 0.05) higher proportion of OCS users, going from 9.0% in the year before to 10.1% the year after the intervention.

### Impact on biological use

The proportion of biological users was 0.22% when analyzing biological use in the year after the reference date among difficult-to-control 5–40-year-old asthma patients who had a PC intervention. In chronic ICS patients aged 5–40 years who never had an intervention (control group) on the other hand, the proportion of biological use was 0.30%. This indicates a 0.08% (95% CI -0.36,0.23%; *p* > 0.05) lower absolute proportion of biological use in the study population receiving at least the first interview compared with a similar, chronic ICS user group never receiving this PC intervention.

## Discussion

The results of this real-world observational study indicate that PC intervention improves adherence to ICS controller therapy and asthma control in difficult-to-control asthma patients.

Our findings of an improved adherence to ICS and higher CTT ratio after PC intervention when using real-life data, is supporting earlier trial results on community pharmacy interventions [9, 21, 22]. Only one trial also focused primarily on patients with difficult-to-control asthma [23]. The PC intervention in our study included an interview in which the community pharmacist assessed the patient’s disease control, knowledge and expectations, inhaler technique and adherence [14]. A follow-up interview was offered routinely, but only occurred for 17% of patients. Multiple interventions did lead to a higher increase in ICS usage, which is in line with studies showing follow-up led to improved outcomes [24, 25]. Adherence is of major importance because it will highly influence the expected pharmacological effect of that drug and the ability to treat the patient’s disease [6, 26]. Besides better adherence, the observed increase in CTT ratio also reflects improved asthma control [17]. An earlier observational study found an 0.1-unit increase in the ratio to result in a significant risk reduction of asthma exacerbations [27]. A higher CTT ratio is also associated with better asthma quality of life [17].

The findings that PC interventions improved medication adherence and asthma control highlight the increasingly important role of the pharmacist in improving patients’ adherence in the healthcare system. Indeed, they may be considered as guardians of adherence in patients with chronic conditions. Their role in adherence support may be fulfilled with or without novel technologies, such as telemedicine [28], and has the potential to amplify their impact on patients health outcomes.

Regarding reliever use, the number of SABA users was found to be reduced in the pharmacist intervention group compared to their own reference period. These real-life observations are also in line with RCTs observing a decline in reliever medication use in the intervention arm [9, 23]. This observed decline in reliever medication likely reflects the desirable improvement in asthma control. In the literature, excessive use of SABA has been associated with insufficient controlled asthma, health-related costs and ultimately a higher mortality risk [29, 30]. It has been shown that a significant number of these health problems could be avoided. Appropriate use of maintenance medication is key in successful asthma management and optimal control reduces the need for reliever medication. In addition, the role of community pharmacies in supporting the asthma management plan was confirmed in a recent study [31].

As the severity of asthma increases, the patient may need OCS therapy more often to adequately manage the disease and associated exacerbations [15, 32]. The percentage of OCS users in our study population of 9–10% was much lower than found in a recent review about difficult-to-treat and severe asthma patients reporting OCS use ranging from 45 to 90% over 1 year [5]. This can be explained by some differences with these studies such as the age difference between our study population aged 5–40 years and several studies with a mean age above 50 years, use of self-reported OCS use and a focus on patients with severe asthma, which is a ‘truly severe’ subgroup of around 20% of the difficult-to-treat patients [15].

Moreover, the total percentage of biological users among the studied difficult-to-treat asthma patients was low during the entire follow-up period. This can partially be explained by the fact that omalizumab was the only reimbursed biological available on the Belgian market until the end of 2016. This means that it was being implemented in common pulmonary practice in 2017. The low observed use of these add-on drugs is also in line with their proven efficacy within a very specific target group of very severe asthma, the high costs and under-studied long-term side effects in a young to adult population [33]. Another reason for the low number can be explained by the fact that some biologicals need to be administered in hospital setting and BelPharData does not capture hospital pharmacy claims. It is important to avoid adding biologicals to the treatment regimen of all difficult-to-control asthma patients. If the inhaler technique and adherence are optimized according to GINA guidelines, the vast majority of asthma patients in the general population can be treated with conventional therapy [4, 34].

Even though the changes we observed were rather small, they might be of greater clinical importance than the numbers reveal. First, a small decrease in antibiotic use may contribute to a reduction in antibiotic resistance. Furthermore, every reduction in SABA use, reflecting better asthma control, might lead to a reduction in exacerbations, hospitalisations and so a lowering of healthcare expenses and potentially deaths. Although an apparently small absolute decline of 0.08% was observed in the proportion of biological users in cases compared with controls, avoiding unnecessary and potential ineffective initiation of biologicals may reduce substantial drug costs [35]. This is the main objective of asthma management in this target population. By improving adherence to controller medication, the group of difficult-to-control asthma patients is reduced to a small group of patients with truly severe asthma where biologicals can be considered.

The pharmaceutical care intervention in Belgium may have benefited from a dense network of pharmacies per inhabitants compared to other countries, adequate training and a renumerated service [10]. Moreover, the PC intervention included counselling to enhance knowledge about asthma and medications, motivation (adherence) and behavioural skills (inhaler technique), all of which are important for clinical promotion and application according to a recent meta-analysis [11]. Notwithstanding the meaningful increase in ICS adherence, the overall effects were modest leaving room for further improvement of the intervention. Our analysis and a previous qualitative study of Fraeyman et al. showed that only a minority of patients received a follow-up interview [14]. Several practical barriers have been reported regarding the implementation of the PC intervention in practice including low familiarity of patients with these kind of services, time pressure, administration regarding the informed consent (signature) and follow-up records [14].

Another strength of this research is the large number of patients using ICS therapy registered in the database. This provided a recent and general picture of the current asthma burden on a national, Belgian level. In addition, the database contains extensive information that offered the opportunity to observe various factors related to disease control. This included objective medication data, such as the use of reliever medication, medications treating exacerbations and add-on drugs. Additionally, using the BelPharData had the great advantage of observing a large amount of PC interventions and patient data in the general population. This database also created the opportunities to perform longitudinal analyses of unidentified asthma patients and their corresponding drug profile. The well-considered study design made it possible to perform an intra-patient comparison, taking their own period of time before the PC intervention as reference, and to compare between patients receiving and not receiving the pharmacist intervention, to account for possible time-trend bias.

An important limitation of this study was the difficulty to clearly identify asthma patients, especially for a control group, since a medical diagnosis of a physician is not being shared with the community pharmacist in the Belgian healthcare system. The control patients used for the analysis of biologicals never received an intervention, meaning that there was no pharmacist assessment as to whether these ICS users were actually patients with asthma. These probably different patient groups make comparative research challenging. Another limitation is ICS usage as a measure of adherence. We assumed that an increase in ICS usage reflected improved adherence through the PC intervention, but theoretically it could also be due to the need for a higher ICS dose in uncontrolled disease. Moreover, the observed differences in proportion of users of asthma-related drugs were rather small. This may be caused by a lower number of patients in the study population by limiting the definition to only difficult-to-control asthma patients among chronic ICS users aged 5–40 years. Another result of defining our study population this way is that patients not diagnosed, recognized or treated as having asthma, are not captured through our definition. In addition, there was no control over the quality of the way the intervention was carried out, since this was a real-life observation. Although, participating pharmacists were offered training, there is undoubtedly a difference in the way pharmacists conduct the counseling interviews and how much time and effort they had invest in it. Nevertheless, the study results suggest that an intervention generally contributes to an average improvement in the asthma patients’ health. Since we had only data from independent pharmacies, our results cannot be generalized to chain pharmacies. Further research is needed to investigate possible differences between rural and urban pharmacies. Finally, there are some limitations on using CTT ratio as a measure for adherence and asthma control. First, a short-acting beta-agonist might be used before exercising, which reflects a good health and disease control. This will overestimate the use of reliever medication taken for poor asthma control and so underestimate the CTT and asthma control. Second, there will be asthma patients on low dose ICS combined with the LABA formoterol as ‘maintenance and rescue therapy’. This would mean that the ICS and LABA do not always reflect good asthma control, since they could also be used as relievers, but would sometimes have been misclassified as controllers when calculating a CTT ratio. Since 2019, GINA guidelines recommend all individuals with asthma receiving ICS-containing controller treatment for mild asthma, to receive as-needed low dose ICS combined with the LABA formoterol [4, 36]. However, this was not yet the case in 2017, when our data were collected.

Future studies could evaluate whether PC intervention also improves therapy adherence and disease control of middle-aged and older adults with asthma and/or COPD. Sharing medical diagnosis might help community pharmacists to identify these patients.

In summary, the results of this real-world observational study indicate that PC intervention improves adherence to ICS controller therapy and asthma control in difficult-to-treat asthma patients. This study suggests that community pharmacist counseling benefits the management of patients’ asthma on a national level and supports a follow-up interview, especially in all difficult-to-control asthma patients.

## Supplementary Information

**Additional file 1.**

## Data Availability

The data that support the findings of this study are available from the Association of Pharmacists Belgium (APB) but restrictions apply to the availability of these data, which were used under license for the current study, and so are not publicly available. Data are however available from the authors upon reasonable request and with permission of APB.
