# Supplementary material for: Community pharmacist counseling improves adherence and asthma control: a nationwide study
Source: BMC Health Serv Res. 2022 Jan 26;22:112. doi: 10.1186/s12913-022-07518-0 (PMC8790878; doi:10.1186/s12913-022-07518-0)
Supplement: Supplementary file 1 — Additional file 1. [file 12913_2022_7518_MOESM1_ESM.docx]

# Additional files

**
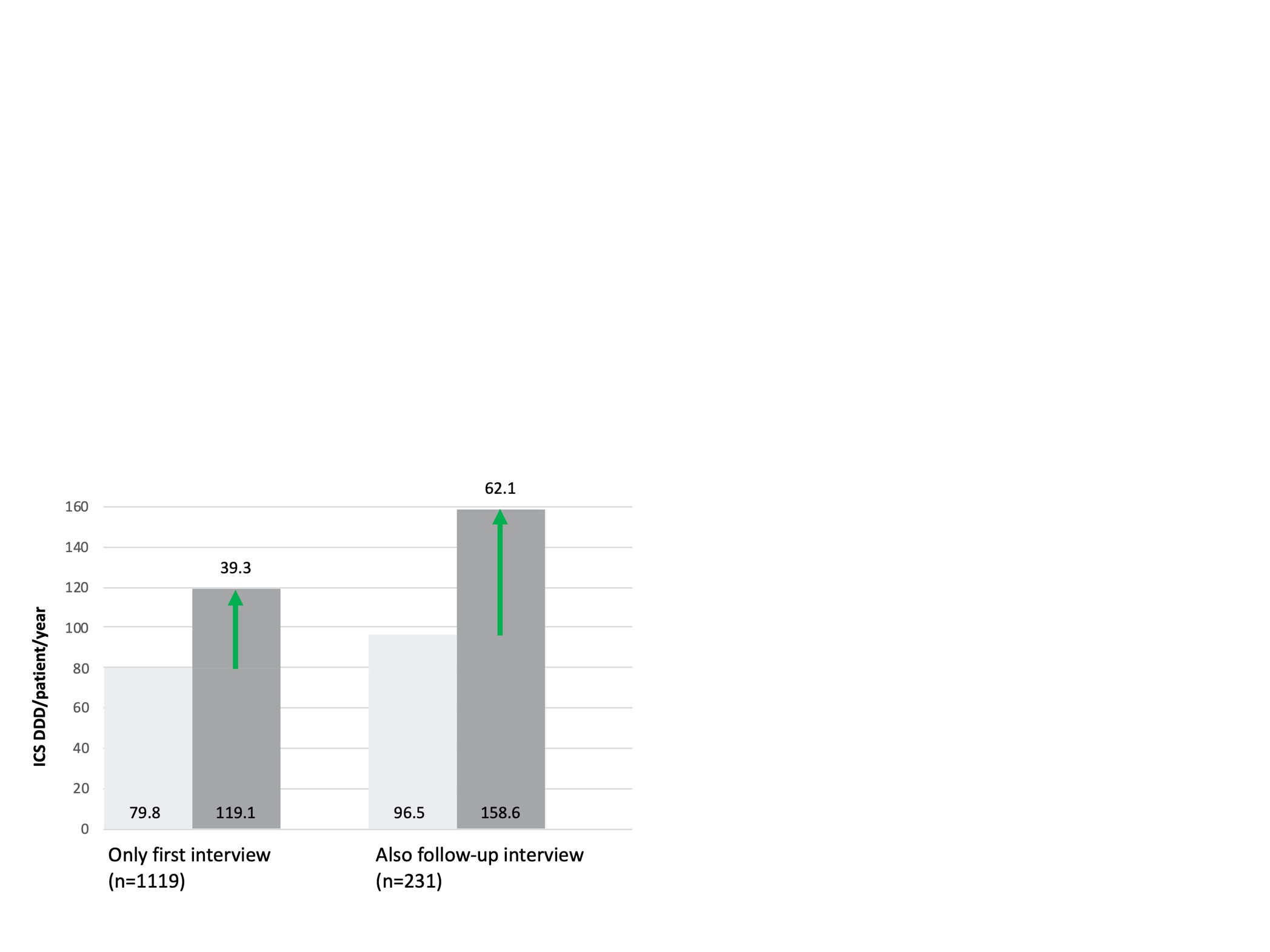
**

**Additional Figure 1.** **ICS usage pre/post intervention among difficult-to-treat asthma patients stratified by number of pharmaceutical counseling (PC) interventions.** The subgroup of patients who received only the first interview (n=1119) showed an increased usage of 39.3 DDD per patient. Additionally, the subgroup of patients who also received a follow-up interview (n=231) showed an even greater gain in ICS usage of 62.1 DDD per patient.

**Additional Table 1. The most commonly used drugs for the treatment of asthma are subdivided by chemical subgroup, active substance and ATC code.**

| **Group** | **Active substance** | **ATC code** |
| --- | --- | --- |
| **Antibiotics** | Antibacterials for systemic use | J01 |
| **Biologicals** | Omalizumab | R03DX05 |
| **Biologicals** | Mepolizumab | R03DX09 |
| **Biologicals** | Benralizumab | R03DX10 |
| **ICS** | Salmeterol and fluticasone | R03AK06 |
| **ICS** | Formoterol and budesonide | R03AK07 |
| **ICS** | Formoterol and beclometasone | R03AK08 |
| **ICS** | Vilanterol and fluticasone furoate | R03AK10 |
| **ICS** | Formoterol and fluticasone | R03AK11 |
| **ICS** | Vilanterol, umeclidinium bromide and fluticasone furoate | R03AL08 |
| **ICS** | Formoterol, glycopyrronium bromide and beclometasone | R03AL09 |
| **ICS** | Beclomethasone | R03BA01 |
| **ICS** | Budesonide | R03BA02 |
| **ICS** | Fluticasone | R03BA05 |
| **LABA/LAMA** | Indacaterol, formoterol or olodaterol and/or aclinidinium, glycopyrronium, tiotropium or umeclidinium | R03BB04-07, R03AL04-06 |
| **OCS** | Methylprednisolone | H02AB04 |
| **SABA/SAMA** | Salbutamol | R03AC02 |
| **SABA/SAMA** | Fenoterol and ipratropium bromide | R03AL01 |
| **SABA/SAMA** | Salbutamol and ipratropium bromide | R03AL02 |

ATC, anatomical therapeutic chemical; ICS, inhaled corticosteroids: LABA, long-acting beta-agonists; LAMA, long-acting muscarinic antagonists; OCS, oral corticosteroids; SABA, short-acting beta-agonists; SAMA, short-acting muscarinic antagonists.
